# Vertical Transmission of COVID-19 to the Neonate

**DOI:** 10.1155/2020/8460672

**Published:** 2020-11-12

**Authors:** Sindy C. Moreno, Justin To, Hajoon Chun, Ivan M. Ngai

**Affiliations:** Obstetrics and Gynaecology Department, Flushing Hospital Medical Centre, Flushing NY, USA

## Abstract

**Objective:**

To estimate the incidence rate of vertical transmission of coronavirus disease 2019 (COVID-19) to the neonate during the third trimester. *Study Design*. We conducted a retrospective observational study of pregnant women diagnosed with COVID-19 during the third trimester, who delivered at Flushing Hospital Medical Centre (FHMC) or Jamaica Hospital Medical Centre (JHMC) between March 20, 2020, and April 30, 2020. The study participants were symptomatic pregnant women diagnosed with COVID-19 via positive SARS-CoV-2 RNA, real-time reverse transcription-polymerase chain reaction (SARS-CoV-2 rRT-PCR) test. Evidence of vertical transmission was assessed in the neonate via a SARS-CoV-2 rRT-PCR test, with nasopharyngeal swab samples collected on the neonates after 24 hours of birth. The exclusion criteria for this study were maternal or neonate records without SARS-CoV-2 rRT-PCR test results, neonates not delivered at FHMC or JHMC, and foetuses with suspected foetal anomalies or incomplete medical records.

**Results:**

We identified 19 symptomatic pregnant women diagnosed with COVID-19, including two women with twin pregnancies. Seven patients (36.8%) were delivered via cesarean. 12 patients (63.1%) presented in spontaneous labour, and 8 (38.1%) had preterm delivery. No maternal intensive care unit admission, maternal sepsis, or maternal mortality was observed. Twenty-one neonates were evaluated for COVID-19 after birth. SARS-CoV-2 rRT-PCR test results were negative in 100% of the neonates. Thirteen neonates (61.9%) were admitted to the neonatal intensive care unit. Prematurity was the most common cause of NICU admission 6 (46.1%), with a length of stay of 5.5 ± 6.4 days. No invasive mechanical ventilation, neonatal sepsis, or neonatal mortality was observed.

**Conclusion:**

In our cohort, symptomatic COVID-19 during the third trimester of pregnancy was not associated with vertical transmission to the neonate.

## 1. Introduction

Coronavirus disease 2019 (COVID-19) is a highly infectious disease that was declared a pandemic by the World Health Organisation (WHO) on March 11, 2020 [1]. The first case of COVID-19 was reported in Wuhan, China [1]. Since late 2019, widespread transmission of COVID-19 has been reported in every country [2]. The United States has the highest number of COVID-19 cases to date: 6,562,390 and total deaths reaching 200,275. NYS currently has the highest COVID-19 incidence [3]. The borough of Queens, one of the most ethnically diverse urban areas in the world, has become an epicentre of COVID-19 with 70,824 cases and total deaths reaching 7,245 to date [4].

Maternal and neonatal data of pregnant women with COVID-19, including vertical transmission, remains limited at the moment [5–10]. Two recent research letters reported three neonates with elevated IgG and IgM antibodies to SARS-CoV-2 born to confirmed COVID-19 pregnant woman despite negative SARS-CoV-2 RNA real-time reverse transcription-polymerase chain reaction (SARS-CoV-2 rRT-PCR) test results, raising the possibility of vertical transmission [11, 12]. However, many of these cases are suspected of postnatal infection after contact with COVID-19-positive parents or caregivers ^5,11,12^. One of the largest series of 116 patients reported no vertical transmission of COVID-19 to the neonates during the third trimester; however, this included the person under investigation without confirmation of COVID-19 testing [10]. Given the importance of vertical transmission and the variability in prior study [5–10] testing and results, our study objective was to determine the incidence of vertical transmission of COVID-19 to the neonate in symptomatic pregnant women with only rRT-PCR laboratory-confirmed COVID-19 in the third trimester.

## 2. Materials and Methods

This was a retrospective observational study of symptomatic women diagnosed with COVID-19 during the third trimester of pregnancy who delivered at Flushing Hospital Medical Centre (FHMC) or Jamaica Hospital Medical Centre (JHMC) between March 20, 2020, and April 30, 2020. All pregnant women who presented to FHMC or JHMC labour and delivery units with signs or symptoms concerning for COVID-19 had nasopharyngeal swab samples taken after obtaining NYS Department of Health (NYSDOH) authorisation. The results were available by the following day. COVID-19 status was diagnosed via a SARS-CoV-2 rRT-PCR test, performed by the NYC Public Health Laboratory. Positive patients were then followed until delivery. Obstetrical management was provided by the standard of care. Evidence of vertical transmission was assessed in the neonate via a SARS-CoV-2 rRT-PCR test with nasopharyngeal swab samples collected on the neonates after birth at FHMC or JHMC. The neonatal SARS-CoV-2 rRT-PCR test was also performed by either the NYC Public Health Laboratory, the FHMC laboratory, or the JHMC laboratory. All sample collection, processing, and laboratory testing complied with WHO guidance [13].

The inclusion criteria were symptomatic pregnant women diagnosed positive for COVID-19 via the SARS-CoV-2 rRT-PCR test in the third trimester and all neonates with complete COVID-19 testing and delivery data. The exclusion criteria for this study were maternal or neonate records without SARS-CoV-2 rRT-PCR test results, neonates not delivered at FHMC or JHMC, and foetuses with suspected foetal anomalies or incomplete medical records. This study was considered exempt by the Institutional Review Board at both institutions.

Maternal demographic data collected included maternal age, ethnicity, gravity, parity, gestational age at delivery, and medical comorbidities such as hypertensive disorders, diabetes, asthma, smoking, and body mass index (BMI), BMI > 30 kg/m^2^. The clinical and laboratory characteristics of women diagnosed with COVID-19 included signs and symptoms such as cough, shortness of breath, gastrointestinal symptoms, fever, highest temperature documented as per complete blood count (CBC), temperature of 38°C or greater, and maternal serum laboratory tests including white blood cell count, lymphocyte count, platelet count, and chest X-ray results. Pregnancy outcomes included mode of delivery, indication for delivery, EBL, and maternal complications due to COVID-19 infection such as supplemental oxygen therapy, invasive mechanical ventilation, intensive care unit (ICU) admission, maternal sepsis, and maternal mortality. Neonatal outcomes included birth weight, APGAR score, neonatal intensive care unit (NICU) admission, and NICU length of stay. Neonatal complications included transient tachypnoea of the neonate (TTN), respiratory distress syndrome (RDS), invasive mechanical ventilation, sepsis, and neonatal demise.

The variables were presented as the mean and standard deviation for continuous and normally distributed variables, by medians with interquartile ranges for nonparametric continuous, and by number and percent for categorical variables.

The primary outcome was the incidence rate of vertical transmission of COVID-19 to the neonate when maternal infection occurred during the third trimester.

## 3. Results

**Figure 1:**
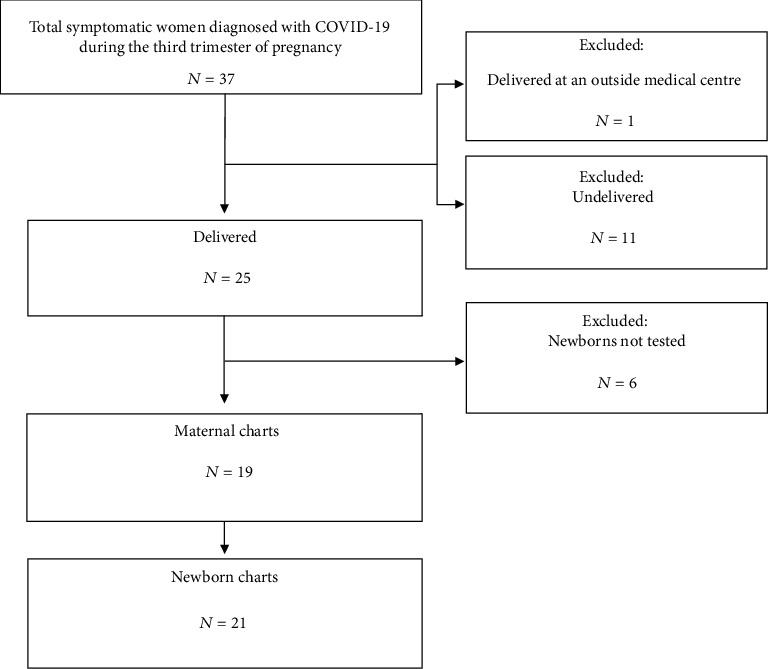
Flowchart of patients included in the analysis.

A total of thirty-seven symptomatic women were diagnosed with COVID-19 during the third trimester of pregnancy at FHMC or JHMC in the period from March 20, 2020, to April 30, 2020. We excluded twelve women: 11 are currently pregnant and 1 woman delivered at an outside medical centre. Six neonates had records without SARS-CoV-2 rRT-PCR test results after birth; thus, the mothers and neonates were also excluded. Nineteen women met the inclusion criteria, including two sets of dichorionic diamniotic twin pregnancies. Twenty-one neonates were included for final analysis (Figure 1).

The majority of the patients were Hispanic 13 (68.4%). Fifteen patients (78.9%) were obese with a BMI ≥ 30 kg/m^2^. Seven patients (36.8%) had additional medical comorbidities including hypertensive disorders 2 (10.5%), diabetes 3 (15.8%), and asthma 2 (10.5%) (Table 1).

Patients diagnosed with COVID-19 presented with different symptoms, including cough (*n* = 19, 100%), fever (*n* = 7, 36.8%) (ranging from 38°C to 38.6°C), and shortness of breath (*n* = 5, 26.3%). Abnormal radiologic chest X-ray findings were present in 68.4% (*n* = 13) of patients, including 11 patients with bilateral infiltrates (84.6%) and 2 patients with unilateral infiltrates (15.4%). The majority of patients presented in spontaneous onset of labour (*n* = 12, 63.1%). Seven patients (36.8%) were delivered via a cesarean section; four of them (57.1%) had a repeat cesarean delivery because of labour. No cases of maternal ICU admission, sepsis, postpartum hemorrhage, or maternal mortality were observed (Table 2).

SARS-CoV-2 rRT-PCR test results were negative in 100% of the neonates. Preterm delivery before 37 weeks was 8 (38.1%), with gestational ages all equal to or greater than 32 weeks of gestational age. Thirteen neonates (61.9%) were admitted to the neonatal intensive care unit (NICU). Prematurity was the most common cause of NICU admission (*n* = 6, 46.1%), with a length of stay of 5.5 ± 6.4 days, with two neonates currently in the NICU due to prematurity of less than 34 weeks. APGAR scores at 1 minute were 8.8 ± 0.7 and at 5 minutes were 8.9 ± 0.2. Low birth weight < 2500 g was identified in seven neonates (33.3%). No invasive mechanical ventilation was required. No neonatal sepsis or neonatal mortality was observed (Table 3).

## 4. Discussion

The possibility of COVID-19 vertical transmission has been a significant concern in symptomatic pregnant women, assuming a high-level of viraemia after symptom onset [14]. Our study shows that there seems to be minimal to no risk for vertical transmission to neonate in maternal patients who test positive for COVID-19 during the third trimester of pregnancy, as 100% of our at-risk neonates for COVID-19 had SARS-CoV-2 rRT-PCR negative results. This suggests that no vertical transmission of COVID-19 to the neonate occurs during the third trimester, given the timing of the pandemic in the United States. Our study supports previous case series that similarly reported no vertical transmission between symptomatic COVID-19-positive pregnant women to their neonates ^6-10^. Our study will be one of the largest cohorts to report no vertical transmission to the neonate from symptomatic with rRT-PCR laboratory-confirmed COVID-19 in the third trimester. Of note, six neonates of COVID-19-positive mothers were excluded from our analysis due to new testing criteria by the NYSDOH after April 30, 2020, and therefore, the neonates did not qualify for testing at the time of birth. However, none of these neonates developed respiratory distress or sepsis or were admitted to the NICU, and no readmissions were observed after discharge from the hospital. Due to these changes by NYSDOH, the remaining eleven undelivered neonates would have been excluded from COVID-19 rRT-PCR testing also at the time of birth.

All our patients were diagnosed with COVID-19 via rRT-PCR, which is a real-time reverse transcription-polymerase chain reaction test for the qualitative detection of nucleic acids from SARS-CoV-2 in upper respiratory specimens and is currently the gold standard for testing for COVID-19 [13]. A recent retrospective analysis in adults showed that the sensitivity of rRT-PCR is 71% for SARS-CoV-2 [15]. Yan et al. published in April 2020 a series of 116 patients with no vertical transmissions of COVID-19 to the neonates during the third trimester; however, this study only included diagnosed COVID-19 patients, who had no confirmatory test results by rRT-PCR [10]. Fang et al. conducted a systematic review and meta-analysis of forty-one neonates in China which reported no clinical signs or symptoms of vertical transmission on the neonates, in which the symptomatic mothers for COVID-19 did not have rRT-PCR performed to confirm COVID-19 infection [16].

Preterm birth was found to be the most common adverse pregnancy outcome in patients with COVID-19. In our cohort, the rate of preterm delivery before 37 weeks was 38.1% (*n* = 8). This is certainly a significant and clinically relevant finding when compared to the incidence of preterm delivery in the United States, estimated at 9.9% [17]. Di Mascio et al. conducted a systematic review and meta-analysis in symptomatic patients diagnosed with COVID-19, reporting a similar rate of preterm delivery (41.1%) in pregnant patients with COVID-19 as our cohort [16]. This systematic review included preterm delivery < 37 weeks 14/56 patients (25%) and preterm delivery < 34 weeks 11/56 patients (19.6%) [16]. Future studies should include a larger number of patients across multiple centres to establish whether preterm delivery is a true adverse outcome in mothers with COVID-19 infection and should additionally include asymptomatic COVID-19 pregnant women to identify any differences in preterm delivery rates.

The strengths of this study include the same maternal and neonate test via rRT-PCR, which is currently the gold standard for testing for COVID-19. All pregnant women included in our study were symptomatic with only rRT-PCR laboratory-confirmed COVID-19 in the third trimester.

The limitations of this study include the small sample size, and future investigations should include larger, multicentre populations to obtain more definitive conclusions regarding the risk of COVID-19 vertical transmission. Our data sample also did not include COVID-19 infections obtained during the first or second trimester, limiting this gestational age for diagnosis of vertical transmission. Future studies should include pregnancies at all gestational ages. In addition, no vaginal samples were collected to evaluate if COVID-19 was detectable during the time of vaginal delivery. However, twelve patients (63.2%) in our study delivered via vaginal delivery, and SARS-CoV-2 rRT-PCR test results were negative in all neonates. Futures studies should include SARS-CoV-2 RNA on vaginal samples and include direct testing of intrauterine tissue samples such as amniotic fluid, cord blood, placenta, viral load, and other variables to further determine the risk for COVID-19 vertical transmission to the neonate.

## 5. Conclusion

In conclusion, our data provides additional evidence to support that vertical transmission of COVID-19 is extremely low, which may be a point of reassurance when counselling patients.

## Figures and Tables

**Table 1:** Maternal demographics.

	*n* = 19
Maternal age (years)	31.7 ± 6.7
Ethnicity	
Caucasian	0 (0)
African American	1 (5.3)
Asian	5 (26.3)
Hispanic	13 (68.4)
Parity	
Nulliparous	9 (47.4)
Gestational age at delivery (weeks)	37.2 ± 2.8
Maternal comorbidities	
Hypertensive disorders	2 (10.5)
Diabetes	3 (15.8)
Asthma	2 (10.5)
Smoking	0 (0)
BMI (kg/m^2^)	33.2 ± 4.5
Obesity BMI ≥ 30 kg/m^2^	15 (78.9)

Data is presented as the mean ± standarddeviation or *n* (%). BMI: body mass index.

**Table 2:** Pregnancy outcomes.

Variable	*n* = 19
Mode of delivery	
Cesarean section	7 (36.8)
Vaginal delivery	12 (63.2)
COVID-19-related maternal complications	
Oxygen support by nasal cannula	5 (26.3)
Oxygen support by nonrebreather mask	1 (5.2)
Invasive mechanical ventilation	0 (0)
Maternal ICU admission	0 (0)
Maternal mortality	0 (0)

Data is presented as the mean ± standarddeviation or *n* (%). ICU: intensive care unit.

**Table 3:** Neonatal outcomes.

Variable	*n* = 21
Neonatal positive SARS-CoV-2 rRT-PCR test	0/21 (0%)
Preterm delivery	8 (38.1)
≤28 weeks	0 (0)
≤34 weeks	2 (25)
≤37 weeks	6 (75)
Birth weight (g)	2972.8 ± 817.3
APGARat5minutes < 7	0 (0)
NICU admission	13 (61.9)
NICU LOS	5.5 ± 6.4
TTN	6 (28.6)
RDS	1 (4.8)
Invasive mechanical ventilation	0 (0)
IVH	0 (0)
Sepsis	0 (0)
NEC	0 (0)
Neonatal demise	0 (0)

Data is presented as the mean ± standarddeviation or *n* (%). NICU: neonatal intensive care unit; NICU LOS: neonatal intensive care unit length of stay; TTN: transient tachypnoea of the neonate; RDS: respiratory distress syndrome; IVH: intraventricular hemorrhage; NEC: necrotising enterocolitis.

## Data Availability

The data will be available on request through contact to request data from the corresponding author.
